# Feasibility of the lidocaine injection method during esophageal endoscopic submucosal dissection

**DOI:** 10.1002/jgh3.12257

**Published:** 2019-09-06

**Authors:** Tetsuya Yoshizaki, Daisuke Obata, Chise Ueda, Norio Katayama, Yasuhiro Aoki, Norihiro Okamoto, Hiroki Hashimura, Masanori Matsumoto, Megumi Takagi, Seitaro Ikeoka, Ryutaro Yoshida, Kenji Momose, Takaaki Eguchi, Hiroshi Yamashita, Akihiko Okada

**Affiliations:** ^1^ Department of Gastroenterology and Hepatology Osaka Saiseikai Nakatsu Hospital Osaka Japan; ^2^ Department of Gastroenterology and Hepatology Kobe Red Cross Hospital Kobe Japan; ^3^ Department of Gastroenterology and Hepatology Kobe University School of Medicine Kobe Japan

**Keywords:** complications, endoscopic submucosal dissection, esophageal neoplasms, hypotension, lidocaine

## Abstract

**Background and Aim:**

Esophageal endoscopic submucosal dissection (ESD) is often technically difficult due to intraoperative body movements. The level of sedation can be increased to suppress body movements, but this may not be successful in all cases. Using local analgesics for submucosal injection during ESD may aid in conscious sedation. This study evaluated the feasibility of the lidocaine injection method (LIM) during esophageal ESD.

**Methods:**

Twenty‐nine patients with superficial esophageal cancer were enrolled in this study at Osaka Saiseikai Nakatsu Hospital, and 1% lidocaine + 0.4% hyaluronate sodium was injected into the submucosa underneath the lesion during esophageal ESD. The main outcome was body movements that disturbed the procedure.

**Results:**

Most patients were male (90%), with a median age of 70 years (interquartile range [IQR]: 66–75 years old), and the median lesion size was 17 mm (IQR: 12–21 mm). The median injection volume of lidocaine was 70 mg (IQR: 55–79 mg). All lesions were successfully removed en bloc. In all cases, there were no body movements that disturbed the procedure. Regarding adverse events of sedation, five patients (17%) had hypotension, four patients (14%) had bradycardia, and seven patients (24%) had hypoxemia during ESD. Convulsions or arrhythmia as adverse events associated with lidocaine were not observed.

**Conclusions:**

Esophageal ESD with LIM did not cause body movements that disturbed the procedure. LIM may help create a stable conscious sedation method for esophageal ESD.

## Graphical abstract

Esophageal endoscopic submucosal dissection (ESD) is often technically difficult due to intraoperative body movements. Esophageal ESD using the lidocaine injection method (LIM) did not cause body movements that disturbed the procedure in our study. LIM may help create a stable conscious sedation method for esophageal ESD.

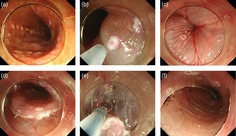

## Introduction

Endoscopic therapy for early esophageal cancer is becoming widely used as a minimally invasive treatment. In particular, endoscopic submucosal dissection (ESD) results in a high en bloc resection rate and accurate pathological diagnosis even for large esophageal cancer.^1^, ^2^, ^3^, ^4^, ^5^, ^6^ However, a high degree of technical skill is required to perform esophageal ESD because the esophagus has a narrow lumen and a thin wall. In addition, unstable endoscopic manipulation due to large body movements, respiratory variation, and esophageal peristalsis makes ESD more difficult. Benzodiazepines, such as midazolam (MDZ), are often used for conscious sedation; however, paradoxically, deeper sedation often increases body movement.^7^, ^8^ Even ESD performed under dexmedetomidine (DEX) or propofol administration may not provide sufficient sedation.^2^, ^3^ Therefore, ESD is often performed under general anesthesia.^5^, ^9^ These problems warrant the development of a technique to overcome the difficulties in effective sedation during esophageal ESD.

Lidocaine is a widely used drug for topical anesthesia. It is used in upper gastrointestinal endoscopy to suppress the pharyngeal reflex. Topical anesthetics have been injected into the submucosal layer for intra‐ or post‐ESD pain in the stomach and colon.^10^, ^11^, ^12^, ^13^ In addition, lidocaine prevents local intestinal spasms.^14^, ^15^, ^16^ These effects of lidocaine may aid in sedation during esophageal ESD. In this study, we evaluated the feasibility of the lidocaine injection method (LIM) during esophageal ESD.

## Methods

### *Patients*

Between January 2017 and June 2018, we treated 35 consecutive patients with early esophageal cancer by ESD at Osaka Saiseikai Nakatsu Hospital. Among them, one patient with synchronous lesions, two patients who underwent ESD with general anesthesia due to a large lesion, and three patients from whom consent to use LIM was not received were excluded from the analysis. Twenty‐nine patients for whom LIM was used during esophageal ESD were enrolled in this study. We confirmed, in interviews before the treatment, that the patients were not allergic to lidocaine. Indications for esophageal ESD were as follows^1^: a lesion diagnosed as high‐grade intraepithelial dysplasia or squamous cell carcinoma at biopsy or^2^ a lesion estimated not to exceed the muscularis mucosae (MM) by white light imaging, narrow band imaging, and iodine staining. The medical records, including age, gender, alcohol consumption, Brinkman index, location of lesions, macroscopic type, details of endoscopic procedure, volume of lidocaine injected, midazolam dosage, adverse events of procedure and sedation, pathological features of the resected lesions, and clinical course after ESD, were retrospectively reviewed. Informed consent about the risks and benefits of ESD and LIM was obtained from all patients. This study protocol was approved by the ethics committee at Saiseikai Nakatsu Hospital.

### *ESD sedation and setting*

All patients underwent local pharyngeal anesthesia for 5 min with 3 mL of 2% lidocaine viscous solution before conscious sedation. DEX (loading dose of 4 μg/kg/h for 5 min; maintenance 0.4 μg/kg/h), MDZ, and 35 mg of petidine hydrochloride were intravenously administered until the sedation level reached 4–5 on the Ramsay sedation scale (RSS). DEX was not used from September 2017 until June 2018. The RSS level was maintained by additional bolus injections of 1 mg of MDZ during ESD. Oxygen saturation (SpO_2_), electrocardiography, pulse rate, and blood pressure were continuously monitored during the procedure. When hypoxia occurred during ESD, oxygen was administered via nasal cannula. GIF‐Q260J (Olympus Co., Tokyo, Japan) with a transparent hood at the tip and carbon dioxide insufflation was used. ESD was carried out using a 1.5‐mm FlushKnife‐BTs (DK2620JBS; Fujifilm, Tokyo, Japan). We used a VIO300D (Erbe Elektromedizin, Tübingen, Germany) electrical generator.

### *ESD procedure and LIM*

**Figure 1:**
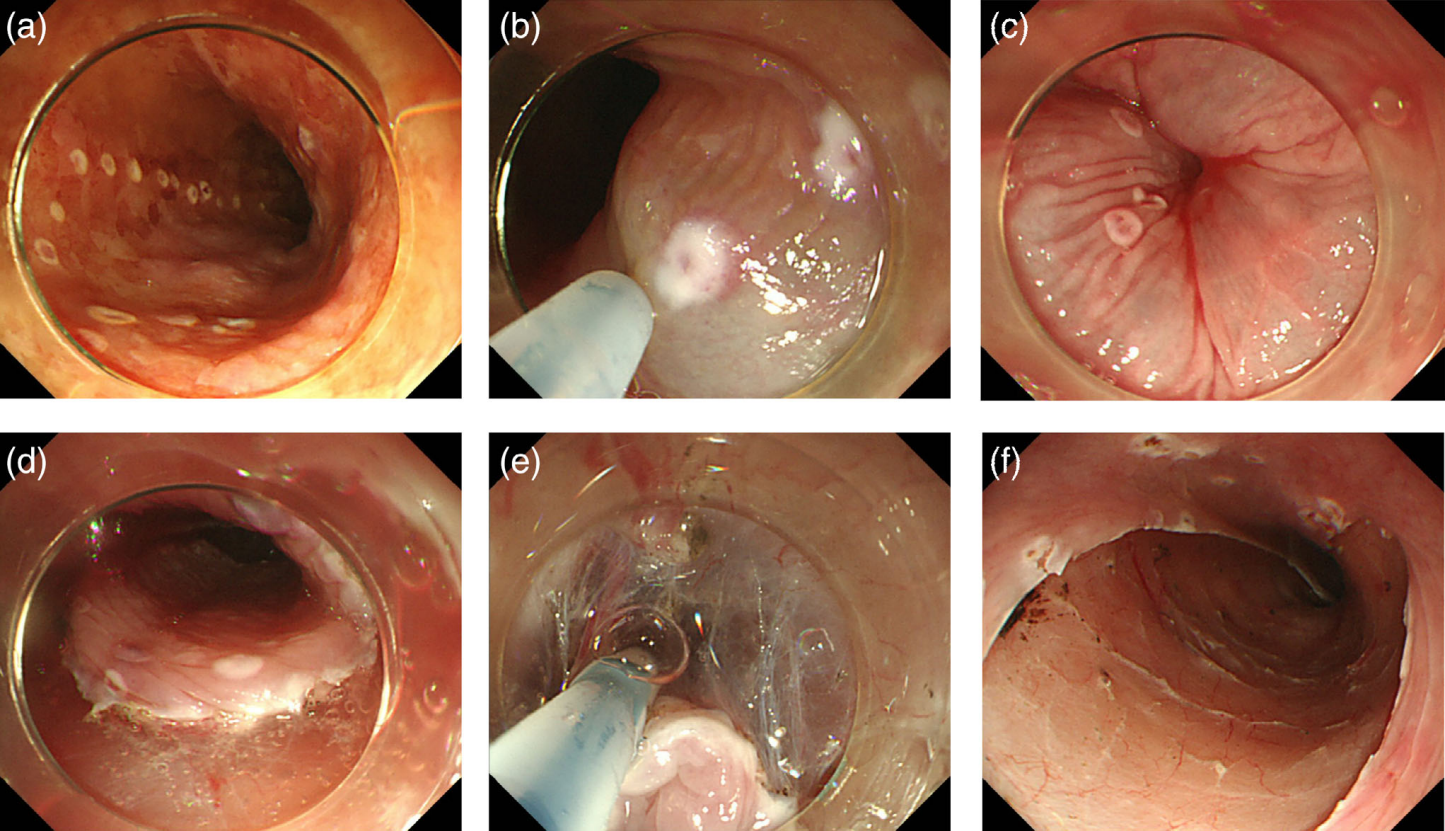
(a) Circumferential marking. (b) 1% lidocaine + 0.4% hyaluronate sodium was injected with an injection needle. (c) After injection. (d) The mucosal incision was started from the oral side. (e) Saline was injected through the FlushKnife. (f) An artificial ulcer after endoscopic submucosal dissection.

An endoscopic overtube (Top Co., Tokyo, Japan) was inserted into the esophagus before ESD. Marking dots were placed outside the iodine‐unstained lesion with a FlushKnife (Fig. 1a); 1% lidocaine + 0.4% hyaluronate sodium (MucoUp; Johnson and Johnson, Tokyo, Japan) was injected into the submucosa using an injection needle (Olympus Co.) (Fig. 1b,c). When the total amount of injected lidocaine exceeded 100 mg, only hyaluronate sodium was injected. The submucosal layer was dissected from the oral side after a mucosal incision on the outside of the marking (Fig. 1d). Saline without lidocaine was used for submucosal injection through the FlushKnife during submucosal dissection (Fig. 1e,f). Hemostasis forceps (Coagrasper, FD‐410LR; Olympus) were used as needed for hemostasis.

### *Histopathological evaluation of the resected specimen*

After ESD, the resected specimen was collected intact, stretched, fastened to a rubber plate using fine needles, and then fixed in 10% buffered formalin solution for 24 h. Each specimen was cut into 2‐mm sections and stained using hematoxylin and eosin. The size, histological characteristics of the tumor, depth of invasion, lymphatic or venous involvement, and presence of tumor at the resection margin were microscopically evaluated.

### *Definition and evaluation*

Body movement was defined as anything that disturbed the procedure. Smoking history was assessed by the Brinkman index (the number of cigarettes per day × years). Hypotension was defined as a systolic blood pressure < 80 mmHg, hypoxemia as an SpO_2_ level < 90%, and bradycardia as a pulse rate < 50 beats/min. Postoperative bleeding was defined as bleeding that required endoscopic hemostatic treatment or cases of massive melena and/or hematemesis with no other apparent source of bleeding. Aspiration pneumonia was defined as the presence of infiltrates and consolidation on chest radiographs after ESD. Perforation was diagnosed by endoscopic findings during ESD or by the presence of pneumomediastinum observed with computed tomography (CT). Postoperative pain was defined as the patient requesting medication for pain relief during hospitalization.

## Results

Characteristics of the patients are summarized in Table 1. Most patients were male (90%), the median alcohol consumption was 72 g/day (interquartile range [IQR]: 42–110 g/day), and the median Brinkman index was 700 (IQR: 0–1200). All lesions were successfully removed en bloc, and there were no adverse events of hemorrhage, perforation, or aspiration pneumonitis. The outcome of sedation is shown in Table 2. In all cases, there were no body movements that disturbed the procedure. The median injection volume of lidocaine was 70 mg (IQR: 55–79 mg). The median total MDZ dosage was 4 mg (IQR: 4–6 mg). Fourteen patients were sedated with MDZ, and 15 patients were sedated with a combination of DEX and MDZ. Regarding adverse events of sedation, five patients (17%) had hypotension, four patients (14%) had bradycardia, and seven patients (24%) had hypoxemia during ESD. No convulsions or arrhythmia as adverse events associated with lidocaine were observed.

**Table 1:** Characteristics of patients

Age, median (IQR), years	70 (66–75)
Gender, *n* (%)	
Male	26 (90)
Female	3 (10)
Alcohol consumption, median (IQR), g/day	72 (42–110)
Brinkman index, median (IQR)	700 (0–1200)
Lesion size, median (IQR), mm	17 (12–21)
Resected specimen size, median (IQR), mm	25 (23–35)
Circumference of the esophageal lumen, *n* (%)	
≥3/4	2 (7)
<3/4	27 (93)
Macroscopic types, *n* (%)	
Elevated	2 (7)
Flat/depressed	27 (93)
Lesion location, *n* (%)	
Cervical esophagus (Ce)	0
Upper thoracic esophagus (Ut)	5 (17)
Middle thoracic esophagus (Mt)	18 (62)
Lower thoracic esophagus (Lt)	5 (17)
Abdominal esophagus (Ae)	1 (3)
Depth of invasion, *n* (%)	
EP/LPM	22 (76)
MM/SM1	4 (14)
SM massive	3 (10)
Treatment time, median (IQR), min	75 (44–95)
En bloc resection, *n* (%)	29 (100)
Adverse events of procedure, *n*	
Postoperative bleeding	0
Perforation	0
Aspiration pneumonia	0

Brinkman index: the number of cigarettes per day × years.

EP, epithelium; IQR, interquartile range; LPM, lamina propria mucosa; MM, muscularis mucosae; SM, submucosa.

**Table 2:** Outcome of sedation

Volume of lidocaine injected, median (IQR), mg	70 (55–79)
Total MDZ dosage, median (IQR), mg	4 (4–6)
Combined use of DEX, *n* (%)	15 (52)
Body movement, *n*	0
Adverse events of sedation, *n* (%)	
Hypotension	5 (17)
Bradycardia	4 (14)
Hypoxemia	7 (24)
Adverse events of lidocaine, *n*	
Convulsions	0
Arrhythmia	0

DEX, dexmedetomidine; IQR, interquartile range; MDZ, midazolam.

The clinical course after ESD is shown in Table 3. Nine patients required medication for pain relief during hospitalization. The median C‐reactive protein (CRP) level on the day following ESD was 0.69 mg/dL (IQR: 0.40–1.09 mg/dL), and the median white blood cells (WBC) count was 8600/mm^3^ (IQR: 6950–9800/mm^3^). One patient developed a fever of over 38°C during hospitalization. In two cases (7%), more than 3/4 of the circumference of the esophageal lumen was resected. Both cases had postoperative stricture and balloon dilatation.

**Table 3:** Clinical course after endoscopic submucosal dissection (ESD)

Postoperative pain, *n* (%)	9 (31)
CRP level after ESD, median (IQR), mg/dL	0.69 (0.40–1.09)
WBC count after ESD, median (IQR), /mm^3^	8600 (6950–9800)
Fever >38°C, *n* (%)	1 (3)
Postoperative stricture	2 (7)

CRP, C‐reactive protein; IQR, interquartile range; WBC, white blood cells.

## Discussion

Intraoperative body movement makes esophageal ESD difficult because the procedure requires delicate endoscopic manipulation in order to avoid muscle layer injury and perforation. In previous studies, intraoperative body movements were suppressed by increasing the level of sedation, such as by MDZ, DEX, or propofol, but even when using these drugs, body movement may not be prevented.^2^, ^3^ Therefore, general anesthesia is often selected to perform ESD without body movement.^5^, ^9^ To the best of our knowledge, this is the first report of LIM during esophageal ESD. When LIM was used for esophageal ESD, there were no body movements that interrupted the procedure, and LIM may be useful for suppressing intraoperative body movements.

Lidocaine is widely used as a local anesthetic. Lidocaine blocks neurotransmission by binding to Na channels, inducing local analgesia. This agent is used in upper gastrointestinal endoscopy to suppress the pharyngeal reflex. The effectiveness of lidocaine during ESD was recently reported.^10^, ^11^, ^12^, ^13^ First, a local injection of lidocaine was used for pain relief when a rectal lesion extending to the dentate line was treated by ESD.^10^, ^11^ In another study, it was reported that submucosal injection of local anesthetic during gastric ESD reduced post‐ESD pain.^12^, ^13^ We believe that the benefit of LIM is the suppression of body movements during ESD by local analgesia. In addition, lidocaine was demonstrated to suppress gastrointestinal peristalsis.^14^, ^15^, ^16^ Nemoto *et al*. reported that lidocaine was effective in suppressing colonic peristalsis.^14^, ^15^ Chen *et al*. reported that esophageal peristalsis was triggered less frequently in response to rapid air distension after intraluminal infusion of lidocaine into the esophagus.^16^ Although the effects of LIM on esophageal peristalsis were not evaluated in our study, we expect that LIM is effective not only for analgesia but also for the suppression of esophageal peristalsis during ESD.

The causes of body movements during ESD are not clear, but one of the factors may be pain. The esophagus has specific nerves that detect mechanical, chemical, or thermal stimuli.^17^ In this study, mechanical receptors within the esophageal wall responded to changes in the local mechanical stresses and strains rather than reacting directly to the luminal pressure. Circumferential stretching is a particularly strong stimulus. During esophageal ESD, esophageal pain may occur due to extensional stimulation by carbon dioxide insufflation to make the treatment field easier to view or due to local mechanical stress by the endoscope against the esophageal wall when dissecting the submucosal layer. In addition, Maeda *et al*. reported that esophageal ESD caused mediastinal emphysema in 63% of patients without perforation.^1^ This suggests that the stimulation by ESD affects the mechanical receptors in the muscular layer and extends beyond the muscular layer because of a lack of esophageal serosa. Esophageal pain may not be completely controlled by intravenous analgesics alone considering that body movements occur even when using intravenous analgesics, such as pethidine and pentazocine, under sedation during esophageal ESD.^2^, ^3^, ^4^ Although there have been no studies evaluating pain during esophageal ESD, esophageal post‐ESD pain was reported in 38.5–58.9% of cases.^6^, ^18^ We speculate that LIM relieves painful stimulation of the esophagus. However, if there is pain other than in the injection area, it may cause poor sedation. For example, discomfort from overtube insertion and excessive air from the endoscope cause body movements, which cannot be prevented by lidocaine.

In addition to lidocaine, other sedatives may help to control body movements during esophageal ESD. Ominami *et al*. reported that 66.2% of patients with esophageal ESD had poor sedation and that this was due to large lesions and alcohol intake.^4^ When propofol was used, body movements were observed in 44% of cases,^2^ and even when DEX and propofol were used in combination, body movements were confirmed in 25% of cases.^3^ On the other hand, increased amounts of sedatives cause a paradoxical response and induce body movements.^7^, ^8^ Therefore, care is needed when adding sedative drugs. However, lidocaine does not cause a paradoxical response because it is a local analgesic. We believe that LIM inhibits body movements in a different manner from sedation in patients with poor sedation.

There are several limitations of the current study. First, this was a single‐center retrospective study. Second, we did not perform a comparison between LIM and no lidocaine injections. The contribution of LIM to the suppression of body movements during esophageal ESD is unknown. Therefore, in the future, a randomized controlled study is required for confirmation of the results of this study.

In conclusion, esophageal ESD with LIM did not cause body movements that disturbed the procedure. LIM may provide a stable conscious sedation method for esophageal ESD.
